# OPTIMA: sensitive and accurate whole-genome alignment of error-prone genomic maps by combinatorial indexing and technology-agnostic statistical analysis

**DOI:** 10.1186/s13742-016-0110-0

**Published:** 2016-01-19

**Authors:** Davide Verzotto, Audrey S. M. Teo, Axel M. Hillmer, Niranjan Nagarajan

**Affiliations:** 1 Computational and Systems Biology, Genome Institute of Singapore, 60 Biopolis Street, Singapore, 138672 Singapore; 2 Cancer Therapeutics and Stratified Oncology, Genome Institute of Singapore, 60 Biopolis Street, Singapore, 138672 Singapore

**Keywords:** Optical mapping, Genomic mapping, Glocal alignment, Overlap alignment, Map-to-sequence alignment

## Abstract

**Background:**

Resolution of complex repeat structures and rearrangements in the assembly and analysis of large eukaryotic genomes is often aided by a combination of high-throughput sequencing and genome-mapping technologies (for example, optical restriction mapping). In particular, mapping technologies can generate sparse maps of large DNA fragments (150 kilo base pairs (kbp) to 2 Mbp) and thus provide a unique source of information for disambiguating complex rearrangements in cancer genomes. Despite their utility, combining high-throughput sequencing and mapping technologies has been challenging because of the lack of efficient and sensitive map-alignment algorithms for robustly aligning error-prone maps to sequences.

**Results:**

We introduce a novel seed-and-extend glocal (short for global-local) alignment method, OPTIMA (and a sliding-window extension for overlap alignment, OPTIMA-Overlap), which is the first to create indexes for continuous-valued mapping data while accounting for mapping errors. We also present a novel statistical model, agnostic with respect to technology-dependent error rates, for conservatively evaluating the significance of alignments without relying on expensive permutation-based tests.

**Conclusions:**

We show that OPTIMA and OPTIMA-Overlap outperform other state-of-the-art approaches (1.6−2 times more sensitive) and are more efficient (170−200 %) and precise in their alignments (nearly 99 % precision). These advantages are independent of the quality of the data, suggesting that our indexing approach and statistical evaluation are robust, provide improved sensitivity and guarantee high precision.

**Electronic supplementary material:**

The online version of this article (doi:10.1186/s13742-016-0110-0) contains supplementary material, which is available to authorized users.

## Background

In recent years, the availability of commercial platforms for high-throughput genome mapping (from, for example, OpGen [1], BioNano Genomics [2] and Nabsys [3] have increased the interest in using these technologies, in combination with high-throughput sequencing data, for applications such as structural variation analysis and genome assembly. In particular, several recent genome assembly projects have highlighted their utility for obtaining high-quality assemblies of large eukaryotic genomes (for example, goat [4] and budgerigar [5] genomes) or studying complex genomic regions [6] and cancer genomes [7]. Mapping technologies typically provide sparse information (an ordered enumeration of fragment sizes between consecutive genomic patterns, for example, restriction sites) for very large fragments of DNA (150 kilo base pairs (kbp) to 2 Mbp) and are thus orthogonal in utility to sequencing approaches that provide base-pair level information for smaller fragments. Combining these two pieces of information therefore requires effective algorithms to align maps to sequences.

Alignment of maps (typically called Rmaps, for restriction maps [8]) to sequences has been widely studied as an algorithmic problem [9] with a range of practical applications, from genome scaffolding [10] to assembly improvement [11] and validation [12]. The general approach has been to translate sequence data to get *in silico* maps and compare these to experimentally obtained maps using dynamic programming algorithms. For large genomes and mapping datasets, naive all-versus-all dynamic programming can be computationally expensive. On the other hand, high error rates in mapping data (optical mapping, for example, can miss one in four restriction sites) has led to the use of model-based scoring functions for sensitively evaluating alignments [13–15]. These often require prior knowledge and modeling of mapping error rates (for example, fragment sizing errors, false cuts and missing cuts) and can be expensive to compute [13, 16, 17]. Alternative approaches with simpler (non-model-based) scoring functions [10] are handicapped by the need to do expensive permutation-based statistical testing to evaluate the significance of alignments, and although recent advances have made this testing more efficient [15], it still scales linearly with genome size. Although these approaches work well for microbial genomes, they typically do not scale well for larger genomes, where they might also have reduced sensitivity. In contrast, commercially available solutions for map-to-sequence alignment (for example, Genome-Builder from OpGen) scale better and have been used for the assembly of large eukaryotic genomes [4] but tend to discard a large fraction of the mapping data (more than 90 %) due to reduced sensitivity and correspondingly lead to increased mapping costs for a project.

Map-alignment algorithms are thus faced with the twin challenges of improving sensitivity and precision on the one hand and reducing computational costs for alignment and statistical evaluation on the other hand. An elegant solution to this problem from the field of sequence-to-sequence alignment is the use of a seed-and-extend approach [18]. However, because maps represent ordered lists of continuous values, this extension is not straightforward, particularly when multiple sources of mapping errors and their high error rates are taken into account [19]. In addition, because error rates can vary across technologies, and even across different runs on the same machine, it is not clear whether a general sensitive map-to-sequence aligner is feasible. An efficient statistical testing framework that helps control for false discovery without prior information about error rates is critical for making such an aligner easy to use and applicable across technology platforms.

In this work, we describe how a sorted search index and the use of a composite seeding strategy can help to efficiently and sensitively detect seed map-to-sequence alignments [20]. Our second contribution is the design of a robust and fast statistical evaluation approach that includes multiple sources of mapping errors in the alignment score and evaluates the significance of the best alignment using all identified feasible solutions. We incorporated these ideas and additional refinements to solve two common alignment problems: glocal alignment, solved with OPTIMA, where an entire map is aligned to a subsequence of a second (typically *in silico*) map; and overlap alignment, solved with OPTIMA-Overlap, where the end of one map is aligned to the beginning of another. When benchmarked against state-of-the-art aligners, OPTIMA and OPTIMA-Overlap typically provide a strong boost in sensitivity (1.6–2 times) without sacrificing precision of alignments (∼99 %). Moreover, our pilot implementations exhibited runtime improvements over commercially available tools (two times faster than OpGen’s Gentig) and orders of magnitude over published, freely available algorithms and software [10, 13].

Finally, these methods are shown to be robust to variations in error distributions while being agnostic to them, suggesting that the methods can deal with different experimental outcomes of the same technology (for example, different map cards or lane types) as well as being applicable across mapping technologies (with minor modifications for pre-processing of data). Because glocal and overlap alignments form the basis of a range of applications that involve the combination of sequence and mapping data (for example, assembly scaffolding, refinement and validation, structural variation analysis and resolving complex genomic regions), OPTIMA and OPTIMA-Overlap can serve as building blocks for these applications, allowing for time- and cost-effective analyses.

### Definitions and problem formulation

**Fig. 1:**
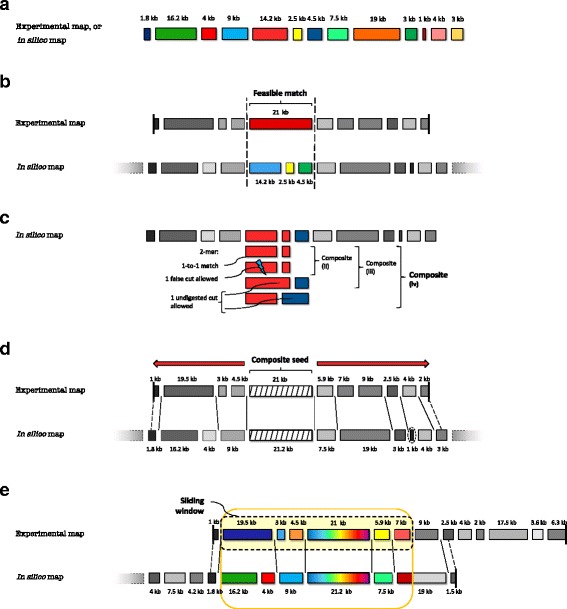
Example of a genomic map and strategies for glocal and overlap map alignment. **a** Example of an experimental or *in silico* map with ordered fragment sizes. **b***Feasible match* within dashed bars (Definition 1). **c***Composite seeds* with *c*=2 (Definition 4), where *Composite (iv)* represents the final composition of seeds with errors used here; the case with one false cut allowed is not directly indexed from the *in silico* maps, but is explored during the seed search process. **d** Seed extension in glocal alignment with dynamic programming (straight lines delimit feasible matches found, dashed lines mark truncated end matches and dashed circles show potentially missing fragments). **e** Sliding-window approach in overlap alignment: for a particular window of fixed size (dashed black border) we first compute a glocal alignment (solid yellow border) from one of its seeds (multicolored box), statistically evaluate it and subsequently extend it until the end of one of the maps is reached on both sides of the seed

High-throughput genome mapping technologies typically work by linearizing large molecules of DNA (for example, in nanochannels [6]) and using enzymes such as restriction enzymes to recognize and label (for example, by cutting DNA) specific patterns throughout the genome (for example, a 6-mer motif). These patterns are then read out (typically, optically) to obtain an ordered set of fragment sizes for each DNA molecule (see Fig. 1a for an example of a map). If corresponding genome sequences or assemblies are available, these can be converted into *in silico* maps through pattern recognition [16].

Let *o*_1_,*o*_2_,…,*o*_*m*_ be the *m* ordered fragment sizes of an experimentally derived map *o* and *r*_1_,*r*_2_,…,*r*_*n*_ be the *n* fragment sizes of an *in silico* map *r*. For simplicity, we suppose here that *m*≤*n* and assume that we can derive standard deviations for *in silico* fragments, that is, *σ*_*j*_ for *r*_*j*_, in a technology-dependent fashion. In an idealized case, we can define the problem of glocally aligning *o* to *r* as a one-to-one correspondence between all the fragments of *o* with a subset of the fragments of *r*, that is, *r*_*l*_,*r*_*l*+1_,…,*r*_*l*+*m*−1_ (we could also reverse the roles of *o* and *r* here). In practice, many sources of errors affect experimentally derived maps, including missing cuts, false/extra cuts, missing fragments, fragment sizing errors and spurious maps [13]. *In silico* maps could also be affected by sequencing or assembly errors [10], but these are less likely to affect alignments because typically they are infrequent. To accommodate errors, we extend the definition of correspondence between map fragments to allow for matches between sets of fragments (see Fig. 1b), as used previously in [10]:

#### **Definition1** (**Feasible match**).

A subset of fragments *o*_*k*_,*o*_*k*+1_,…,*o*_*s*_ aligned as as a whole entity to a subset of *in silico* fragments *r*_*l*_,*r*_*l*+1_,…,*r*_*t*_ is called a *feasible match* if and only if:

(1) $$ \left|{\frac{\sum\limits_{i=k}^{s} o_{i} - \sum\limits_{j=l}^{t} r_{j}}{\sqrt{\sum\limits_{j=l}^{t} {\sigma_{j}^{2}}}}}\right| \le C_{\sigma},  $$

where *C*_*σ*_=3 is an appropriate bound if sizing errors are approximately normally distributed.

#### **Definition2** (**Glocal alignment**).

A valid *glocal alignment* is an ordered set of matches *M*_1_,*M*_2_,…,*M*_*w*_ between experimental and *in silico* fragments, such that all the experimental fragments *o*_1_,*o*_2_,…,*o*_*m*_ are aligned to a subset of the *in silico* fragments *r*_*t*_,*r*_*t*+1_,…,*r*_*v*_, and both sets are orderly partitioned by all the matches *M*_1…*w*_ without overlaps, with *w*≤*m* and *w*≤*v*−*t*+1.

Missing fragments, which usually arise from short fragments below the experimental detection limit (for example, 2 kbp), can be handled in this framework by allowing gaps, that is, with the option of ignoring short fragments for the purpose of the *C*_*σ*_ test (Eq. 1).

#### **Definition3** (**Overlap alignment**).

A valid *overlap alignment* is an ordered set of matches *M*_1_,*M*_2_,…,*M*_*w*_ that allows experimental maps and *in silico* maps to only partially align with each other, with *M*_1_ and *M*_*w*_ each corresponding to an end of one of the maps (for example, Fig. 1e).

In general, because maps can have truncated ends, we relax the *C*_*σ*_ test to be only an upper bound on matches comprising the ends of experimental maps, for example:

$$\sum\limits_{i=k}^{m} o_{i} - \sum\limits_{j=l}^{t} r_{j} \le C_{\sigma} \sqrt{\sum\limits_{j=l}^{t} {\sigma_{j}^{2}}}, $$

or a lower bound on matches at the ends of *in silico* maps, for example:

$$\sum\limits_{i=k}^{s} o_{i} - \sum\limits_{j=l}^{v} r_{j} \ge C_{\sigma} \sqrt{\sum\limits_{j=l}^{v} {\sigma_{j}^{2}}}. $$

## Methods

OPTIMA is the first alignment tool based on the seed-and-extend paradigm that can deal with erroneous mapping data. The basic paradigm is similar to that used for the alignment of discrete-valued sequences (allowing for mismatches and indels) and is as follows. We start by indexing the *in silico* maps, so that we can use this information later, and find seeds for each experimental map *o* corresponding to some indexed regions of those sequences. We then extend these seeds by using dynamic programming to try to align the whole experimental map to the corresponding *in silico* map region. For each map *o*, *feasible* solutions – as defined by the index structure, size of the genome and maximum error rate – are then evaluated by a scoring scheme to select the optimal solution. Finally, the statistical significance and uniqueness of the optimal solution are determined by comparing and modeling all the identified feasible solutions.

### Indexing continuous-valued seeds

**Fig. 2:**
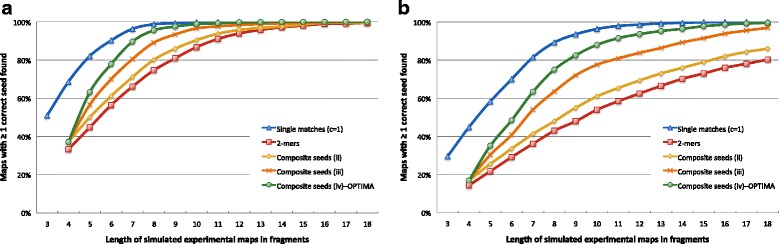
Comparison of sensitivity between different seeding approaches for the human genome. **a** The easier scenario (**a**). **b** The harder scenario (**b**). For each corresponding length in fragments, we report the percentage of maps with at least one correct seed detected (out of 100 maps). Note that the approach used in OPTIMA, *Composite seeds (iv)*, was able to find the correct location for more than 99 and 88 % of maps with at least ten fragments in scenarios (**a**) and (**b**), respectively

The definition of appropriate seeds is critical in a seed-and-extend approach in order to maintain a good balance between sensitivity and speed. A direct extension of discrete-valued seeds to continuous values is to consider values that are close to each other (as defined by the *C*_*σ*_ bound) as matches. However, as mapping data typically have high error rates [13, 16] and represent short sequences (for example, on average, optical maps contain 10–22 fragments, representing roughly a 250 kbp region of the genome), a seed of *c* consecutive fragments (*c*-mer) is likely to have low sensitivity unless we use a naive *c*=1 approach (see Fig. 2 for a comparison) and pay a significant runtime penalty that scales with genome size [14, 16]. Therefore, we propose and validate the following composite seed extension for continuous-valued seeds, analogous to the work on spaced seeds for discrete-valued sequences [21].

#### **Definition4** (**Composite seed**).

*Let *$r_{j_{1}}$, $r_{j_{2}}$ and $r_{j_{3}}$ be *consecutive restriction fragments from a reference* in silico *map. A continuous-valued* composite seed, *for c*=2, *is given by including all of the following:*

- *(i)* the *c*-mer $r_{j_{1}}, r_{j_{2}}$, corresponding to no false cuts in the *in silico* map;
- *(ii)* the *c*-mer $r_{j_{1}}+r_{j_{2}}, r_{j_{3}}$, corresponding to a missing cut in the experimental map (or false cut in the *in silico* map); and
- *(iii)* the *c*-mer $r_{j_{1}}, r_{j_{2}}+r_{j_{3}}$, corresponding to a different missing cut in the experimental map (or false cut in the *in silico* map).

The reference index would then contain all *c*-tuples corresponding to a composite seed, as defined in Definition 4, for each location in the reference map. In addition, to account for false cuts in the experimental map, for each set of consecutive fragments $o_{i_{1}}$, $o_{i_{2}}$ and $o_{i_{3}}$ in the experimental maps, we search for *c*-tuples of the type $o_{i_{1}}, o_{i_{2}}$ and $o_{i_{1}} + o_{i_{2}}, o_{i_{3}}$ in the index (see *Composite seeds (iv)* depicted in Fig. 1c).

To index the seeds, we adopt a straightforward approach where all *c*-tuples are collected and sorted into the same index in lexicographic order by *c*_1_ (where the *c*_*i*_ are elements in the *c*-tuple). Lookups can be performed by binary search over fragment-sized intervals that satisfy the *C*_*σ*_ bound for *c*_1_ and a subsequent linear scan of the other elements *c*_*i*_, for *i*≥2, while verifying the *C*_*σ*_ bound in each case. Note that, because seeds are typically expected to be of higher quality, we can apply a more stringent threshold on seed fragment size matches (for example, we used $C^{Seed}_{\sigma } = 2$).

As shown in the “Results and discussion” section, this approach significantly reduces the space of candidate alignments without affecting the sensitivity of the search. A comparison between the various seeding approaches is shown in Fig. 2, which highlights the advantages of composite seeds with respect to 2-mers.

Overall, the computational cost of finding seeds using this approach is *O*(*m* (log*n*+*c* *#seeds*_*c*=1_)) per experimental map, where *n* is the total length of the *in silico* maps in fragments, *m*≪*n* is the length of the experimental map and *#seeds*_*c*=1_ is the number of seeds found in the first level of the index lookup, before narrowing down the list to the actual number of seeds that will be extended (*#seeds*). The cost and space of creating the reference index is thus *O*(*c* *n*), if the number of errors considered in the composite seeds is limited and bounded (as in Definition 4), and radix sort is used to sort the index. This approach drastically reduces the number of alignments computed in comparison to more general, global alignment searches [10], as will be shown later in the “Results and discussion” section.

### Dynamic programming-based extension of seeds

In order to extend a seed to get a glocal alignment we adopt a scoring scheme similar to that used in SOMA (see [10]). This allows us to evaluate alignments without relying on a likelihood-based framework that requires prior information on error distributions as input [13]. In addition, we can use dynamic programming to efficiently find glocal alignments that optimize this score and contain the seed (see Fig. 1d); specifically, for each seed side we proceed along the dynamic programming matrix by aligning the end of the *s*th experimental fragment with the end of the *t*th *in silico* fragment using backtracking to find feasible matches, that is, those that satisfy Eq. 1 and minimize the total number of cut errors (*#cuterrors*= missing cuts + false cuts + missing fragments found), with ties being broken by minimizing a *χ*^2^ function for fragment sizing errors:

(2) $$ {Score}_{s, t} = \min_{k \le s, l \le t} C_{ce} (s - k + t - l) + {\chi}_{k..s, l..t}^{2} + {Score}_{k-1, l-1},  $$

where the first index of each subscript represents experimental fragments, the second index represents the *in silico* fragments, *s*−*k* is the number of false cuts, *t*−*l* is the number of missing cuts, *C*_*ce*_ is a constant larger than the maximum possible total for *χ*^2^, ${\chi }_{k..s, l..t}^{2} = \left (\sum \limits _{i=k}^{s} o_{i} - \sum \limits _{j=l}^{t} r_{j}\right)^{2} / \left (\sum \limits _{j=l}^{t} {\sigma _{j}^{2}}\right)$ and *Score*_0,0_=0, *Score*_*i*,0_=*∞* and *Score*_0,*j*_=*∞*.

Note that a small *in silico* fragment is considered as missing if this condition allows for a valid alignment that improves the local *χ*^2^ on nearby matches by half (up to three consecutive fragments).

As in [16], we band the dynamic programming and its backtracking to avoid unnecessary computation. In particular, as we show in Supplementary Note 1 in Additional file 1, based on parameter estimates for optical mapping data, restricting alignments to eight missing cuts or five false cuts, consecutively, should retain high sensitivity. In addition, we stop the dynamic programming-based extension if no feasible solutions can be found for the current seed after having analyzed at least *f* fragments (default of five).

The computational cost of extending a seed (*c*-tuple) of an experimental map with *m* fragments is thus, in the worst case, *O*((*m*−*c*) *δ*^3^) time, where *δ* is the bandwidth of the dynamic programming, and *O*((*m*−*c*)^2^) space for allocating the dynamic programming matrix for each side of the seed.

### Statistical significance and uniqueness analysis

**Fig. 3:**
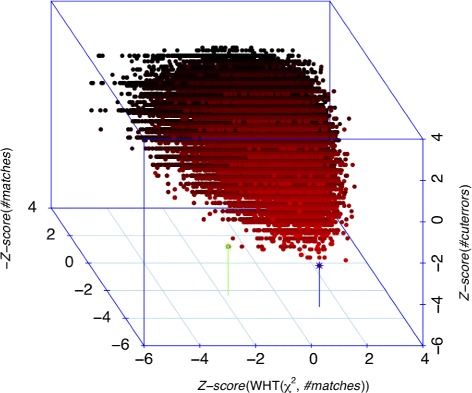
Representation of candidate alignments as a function of alignment features. The results shown are based on aligning a 26-fragment simulated experimental map on the human reference genome. The green comet represents the true solution, and also the best solution *π*^∗^ found by OPTIMA (*p*-value *p*^∗^=2.16e^−9^), while the blue comet belongs to a false alignment with the lowest number of cut errors (*p*=7.35e^−6^). Note here that despite having many near-optimal solutions, OPTIMA unambiguously identifies the correct solution based on its statistical analysis

To evaluate the statistical significance of a candidate alignment, we exploit the fact that we have explored the space of feasible alignments in our search and use these alignments to approximate a random sample from a (conservative) null model. The assumption here is that there is only one true alignment and that, therefore, the population of these sub-optimal alignments can provide a conservative null model for evaluating the significance of an alignment; more specifically, for each candidate alignment found, we compute its distance from the null model in a feature space (to be defined later) using a Z-score transformation and then use this score to evaluate whether it is optimal, statistically significant and unique (see Fig. 3 for an example).

We start by identifying a set *F* of features, independent with respect to false positive (or random) alignments, that are expected to follow the normal distribution (for example, using the central limit theorem) and be comparable between different alignments of the same experimental map, and we compute a Z-score for each feature *f*∈*F* and for each candidate solution π∈Π identified through the seeding method. Each Z-score takes into account the mean and standard deviation of a particular feature *f* among all candidate alignments found:

(3) $$ {Z-score}(\mathrm{\pi} \in \mathrm{\Pi}, f) = \frac{f_{\mathrm{\pi}} - {Mean}(f_{\mathrm{\Pi}})}{{SD}(f_{\mathrm{\Pi}})}.  $$

Accounting for all considered features *f*_*i*_, with 1≤*i*≤*k* and *k*≥2, the resulting score is given by:

(4) $$ \vartheta(\mathrm{\pi} \in \mathrm{\Pi}) = {Z-score}\left(\sum_{i} s_{i} \times {Z-score}(\mathrm{\pi}, f_{i})\right),  $$

where *s*_*i*_=1 if lower values of feature *f*_*i*_ are preferable and −1 otherwise, and the corresponding *p*-value is *p*_π_=Pnorm(*𝜗*(π)), that is, the probability that a ‘random’ Z-score will be less than *𝜗*(π) under the standard normal distribution.

In our case, we chose a set of features based on the number of matches (*#matches*), the total number of cut errors and the Wilson-Hilferty transformation (WHT) of the *χ*^2^ score for sizing errors (which converges quickly to a standard normal distribution):

(5) $$ \textrm{WHT}\left({\chi}^{2}, {\# matches}\right) = \frac{\sqrt[3]{\frac{{\chi}^{2}}{{\# matches}}} - \left(1 - \frac{1}{9} \frac{2}{{\# matches}}\right)}{\sqrt{\frac{1}{9} \frac{2}{{\# matches}}}}.  $$

Note that this set can be shown to be composed of approximately independent features for false positive alignments (Z-score pairwise covariances between all features did not show a significant alteration of the final Z-scores when accounting for them in all of our simulations). By the central limit theorem, the mean of the first two features can be approximated by the normal distribution when the number of candidate solutions is large enough (typically, greater than 60 distinct solutions), and, by Slutsky’s theorem, their sample variance would not have a significant effect on the distribution of the test statistics. As lower values of *#cuterrors* and WHT(*χ*^2^,*#matches*) indicate better solutions, while a higher number of matches represents more reliable alignments, we need to adjust the signs of their Z-scores accordingly. The final Z-score *𝜗*(π) computed for each candidate solution π is therefore given by:

(6) $$ \begin{aligned} \vartheta(\mathrm{\pi} \in \mathrm{\Pi}) &= {Z-score}(-{Z-score}(\mathrm{\pi}, {\# matches})\\ &\quad+ {Z-score}(\mathrm{\pi}, {\# cut errors})\\ &\quad+ {Z-score}(\mathrm{\pi}, \textrm{WHT}({\chi}^{2}, {\# matches}))\,), \end{aligned}  $$

which can be subsequently converted into the *p*-value *p*_π_. The candidate solution *π*^∗^ with the lowest *p*-value *p*^∗^ is reported as the optimal solution, as shown in Fig. 3.

The statistical significance of the optimal solution can then be assessed through a false discovery rate *q*-value analysis [22] based on all candidate solutions found for comparable experimental maps, for example, those with the same number of fragments (default of *q*=0.01). To assess uniqueness, we set a threshold on the ratio of *p*-values between the best solution and the next-best solution (default of five). See Supplementary Note 2 in Additional file 1 for further algorithmic details.

In summary, our statistical scoring approach finds an optimal solution and evaluates its statistical significance and uniqueness in a unified framework, thus allowing for savings in computational time and space compared to a permutation test, without restricting the method to a scenario where experimental error probabilities are known *a priori*.

### Extension to overlap alignment

To extend OPTIMA to compute and evaluate overlap alignments – a key step in assembly pipelines that use mapping data [4, 5, 23] – we use a sliding-window approach based on OPTIMA. This allows us to continue using the statistical evaluation procedure defined in OPTIMA that relies on learning parameters from comparable alignments (that is, those with the same number, size and order of experimental fragments) in a setting where the final alignments are not always of the same length and structure.

Briefly, for each map, OPTIMA-Overlap first finds optimal sub-map alignments, evaluates their significance and uniqueness, and then tries to extend the candidate alignments found until it reaches the ends of either the experimental map or the *in silico* map, in order to choose the most significant overlap alignments (see Fig. 1e). This approach begins by dividing an experimental map into sub-maps of length *l* with a sliding window and then glocally aligning them to *in silico* maps using OPTIMA (again allowing for truncated ends to account for high error rates). Each glocal alignment sub-problem will then return either:

(i) a significant and unique sub-map alignment;
(ii) an alignment labeled as non-significant and/or non-unique (which will be considered as a false alignment); or
(iii) no feasible alignments found.

Optimal solutions to the sub-problems are then ranked by *p*-value (smallest to largest) and iterated through to select sub-maps that should be extended. At each stage we check the significance and uniqueness of the reported solutions (compared to the others), as well as checking for potential cases of identical or conflicting alignments as defined below.

#### **Definition5** (**Conflicting alignments**).

A sub-map alignment *π*_1_ is said to be *conflicting* with another alignment *π*_2_ if either:

- the sub-map of *π*_1_ overlaps the sub-map of *π*_2_; or
- *π*_1_ aligns to the same *in silico* map as *π*_2_, but in a different location or strand.

Conflicting alignments can result in ambiguous placement of an experimental map on a database of *in silico* maps, but condition (a) could be relaxed in some cases, for example, when experimental maps are known to overlap multiple *in silico* maps in the same region. Therefore, while iterating through the list of sub-maps, the following rules are implemented:

1. Significance: if the current solution *π*_*i*_ is labeled as a false alignment, then we stop iterating through the rest of the list.
2. Uniqueness: we skip an alignment if either: (i) *π*_*i*_ represents the same overlap alignment as a more significant solution; (ii) *π*_*i*_ is *conflicting* with a solution with a lower *p*-value (that is, seen before); or (iii) *π*_*i*_ is not unique with respect to other solutions *π*_*j*_ with *j*>*i* (that is, having greater *p*-values) that it is *conflicting* with.
3. Extension with dynamic programming: optimal overlap solutions are identified according to Eq. 2, where ties are broken in favor of longer valid alignments.

This approach allows us to report multiple overlap alignments (including split alignments) for an experimental map while using the *q*-value analysis, as before, to report all alignments with *q*≤0.01. For the *q*-value analysis, we consider all candidate solutions found for the sliding windows in order to learn the *q*-value parameters. In addition, we can reuse the dynamic programming matrix computed for each seed across sub-map alignments and thus complete the overlap alignment with the same asymptotic time and space complexity as the glocal alignment.

## Results and discussion

### Generation of benchmarking datasets

To benchmark OPTIMA and OPTIMA-Overlap against other state-of-the-art map aligners, we first developed synthetic datasets that aim to represent two ends of the spectrum of errors in mapping data for eukaryotic genomes. These scenarios were defined by confidently aligning (using SOMA [10] and manual curation) two sets of maps from different experimental runs for optical mapping (using the Argus system from OpGen) on a human cell line. The first scenario, (A), was defined based on lanes that were reported by the Argus machine to have high quality scores, while the second scenario, (B), was defined by lanes with the lower qualities that were typically obtained on the system. We estimated three key parameters from the data: *d*, the average restriction enzyme digestion rate; *f*_100_, the average false cut rate per 100 kbp; and the fragment sizing errors for predefined (reference) *in silico* fragment size ranges (these were fixed for both scenarios and recorded as relative deviations of the empirical sizes from the reference sizes):

- Easier scenario: *d*=0.78 (corresponding to missing cut rate of 22 %); *f*_100_=0.97; and probability at 0.5 for missing fragments of size below 1.2 kbp, 0.75 below 600 bp and 1 below 350 bp.
- Harder scenario: *d*=0.61 (corresponding to missing cut rate of 39 %); *f*_100_=1.38; and 0.5 for missing fragments of size below 2 kbp, 0.75 below 800 bp and 1 below 350 bp.

For each scenario, we first simulate the map sizes using empirically derived distributions from real maps (average size of approximately 275 kbp, containing 17 fragments) and extract the corresponding reference sub-maps by sampling start locations uniformly from the *in silico* maps (possibly creating truncated end fragments). Then we introduce cut errors using the probability distributions described in [13] with the above parameters, that is: first, we remove missing cuts following a Binomial distribution with probability 1−*d*; next, we insert false cuts modeled as a Poisson process with rate *f*_100_ (avoiding creation of small fragments less than 1.2 kbp in size); and finally, we remove small fragments with the probabilities described above. Sizing errors are introduced by sampling from the empirical errors found for each range of reference fragment sizes. Simulated experimental maps smaller than 150 kbp or with fewer than ten fragments are discarded, mimicking the pre-processing stage on the Argus system.

We generated 100 times greater coverage of maps with errors for the *Drosophila melanogaster* (BDGP 5) and *Homo sapiens* (hg19/GRCh37) genomes using the *Kpn*I restriction pattern GGTAC’C, where the apostrophe indicates the position of the cut, which resulted in 13,920 fragments genome-wide (forward and reverse strands) with an average fragment size (AFS) of 17.3 kbp and 573,276 fragments with AFS = 10.8 kbp, respectively.

### Glocal alignment results

OPTIMA was compared against the state-of-the-art algorithms Gentig v.2 (alignment module) [16, 17, 24], SOMA v.2 [10] and Valouev’s likelihood-based fit alignment [13] for glocally aligning the simulated maps over their respective *in silico* reference genomes. TWIN [19] was not included in this comparison as it does not allow for errors and missing information in experimental maps.

We also ran variations of these algorithms from their default options (d): specifically, by providing the true error distribution parameters used in the simulations as input (tp), the adjusted AFS based on the organism under analysis (a) and parameter values published in the respective papers (instead of the software’s default ones), to provide, in addition, the true error distribution rates (p); and by allowing the trimming of map ends in the alignment (t). Moreover, SOMA [10] was modified to correctly handle missing *in silico* fragments up to 2 kbp, to run only for *C*_*σ*_=3, to make its results comparable, and by inverting the role of *in silico*–experimental input maps (v). We omitted SOMA’s statistical test (also for Valouev’s likelihood method, where it is not enabled by default), because it is unfeasible for large datasets [19], and applied only its uniqueness test (F-test). Further details about the running parameters are provided in Supplementary Note 3 in Additional file 1. OPTIMA alignments were performed on both strands of the *in silico* maps, without trimming end fragments or removing any small *in silico* fragments.

As can be seen from the results in Table 1, OPTIMA reports alignments with very high precision, greater than 99 % in most cases, independent of the genome size and the dataset error rate. In comparison, Gentig has similarly high precision on the *Drosophila* genome but lower precision on the human genome, with as low as 80 % precision under scenario (B) (with default parameters). Without their computationally expensive statistical tests, which can increase the runtime by a factor of greater than 100, SOMA and the likelihood-based method have much lower precision, particularly on the human genome. In addition, in terms of sensitivity, OPTIMA was found to be notably better than other aligners, even when the true error distribution rates were provided as input to these algorithms. In particular, for the higher quality scenario (A), OPTIMA is more than 1.5 times as sensitive as Gentig, and for the commonly obtained scenario (B), OPTIMA is more than twice as sensitive as Gentig.

**Table 1:** Comparison of all methods and their variants on glocal map-to-sequence alignment

Algorithm	*Drosophila* (A)		*Drosophila* (B)		Human (A)		Human (B)	
	S	P	S	P	S	P	S	P
OPTIMA	**90**	**100**	**49**	**99**	**83**	**100**	**43**	**98**
Gentig v.2 (d)	59	**100**	24	**99**	53	96	20	80
Gentig v.2 (tp)	59	**100**	24	98	54	95	20	88
SOMA v.2 (v)	72	73	31	39	50	50	17	20
Likelihood (d+a)	49	49	29	30	24	24	14	14
Likelihood (d+a+t)	64	65	38	39	33	34	18	19
Likelihood (p+a+t)	75	75	39	39	62	62	19	20

**Fig. 4:**
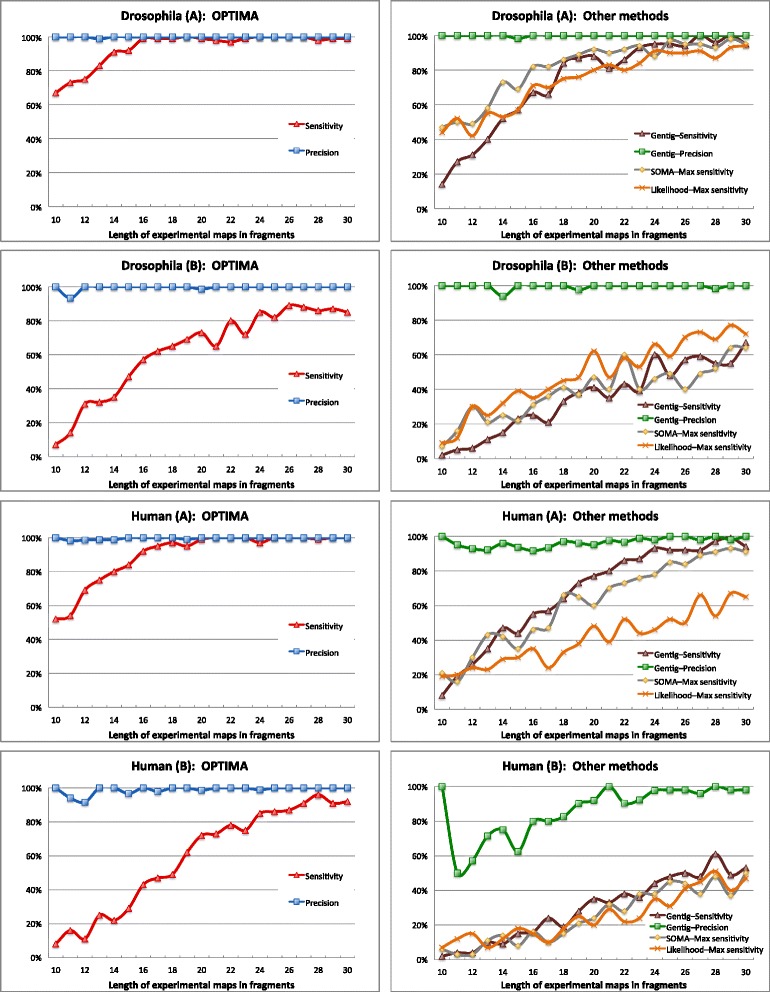
Glocal alignment as a function of the number of fragments in the experimental maps. Gentig results are plotted for setting (d) and likelihood-based fit alignment results are for setting (d+a+t). Results are reported for 100 maps for each bin of simulated datasets for *Drosophila* and human scenarios (**a**) and (**b**)

Sensitivity (S) and precision (P) are percentages and the best values across all methods are highlighted in bold. Results are based on the alignment of a subset of 2100 maps, as used in Fig. 4

These results are further broken down in Fig. 4 as a function of the number of fragments in the experimental maps, showing that OPTIMA uniformly achieves more than twice the sensitivity for the smaller maps that are frequently obtained in real datasets. The relatively higher sensitivities of SOMA and the likelihood-based method in these experiments are likely to be artifacts of relaxed settings in the absence of their statistical tests. These results highlight OPTIMA’s high precision and improved sensitivity across experimental conditions and suggest that it could adapt well to other experimental settings.

In Table 2, we further compare all methods on their running time and worst-case complexity (runtime and space). It can be seen that SOMA and the likelihood-based methods are at least an order of magnitude slower than OPTIMA and Gentig. Gentig’s proprietary algorithm is based on work that has been previously published [17, 24], but its current version uses an unpublished hashing approach. In comparison, OPTIMA is two times faster while being more than 50 % more sensitive than Gentig.

**Table 2:** Running time and worst-case complexity for various glocal map-to-sequence aligners

Algorithm	Complexity		Running time	
	Time	Space	*Drosophila*	Human
OPTIMA	*O*((*m*−*c*) *δ*^3^ *#seeds*)	*O*((*m*−*c*)^2^+*c* *n*)	**54 m**	**36 days**
Gentig v.2 (d)	*O*(*#it* *m* *δ*^3^ *#hashes*)	*O*(*m*^2^+*n*+|*HashTable*|)	1.32 h	75 days
Gentig v.2 (tp)			1.85 h	174 days
SOMA v.2 (v)	*O*(*m*^2^ *n*^2^)	*O*(*m* *n*)	1.28 years	1,067 years
Likelihood (d+a)	*O*(*m* *n* *δ*^2^)	*O*(*m* *n*)	22.22 h	2.72 years
Likelihood (d+a+t)			19.62 h	2.38 years
Likelihood (p+a+t)			41.73 h	5.53 years

Running times reported are estimated from 2100 maps and extrapolated for the full datasets (82,000 *Drosophila* maps and 2.1 million human maps, for 100 × coverage; single-core computation on Intel x86 64-bit Linux workstations with 16GB RAM). The best column-wise running times are reported in bold. Note that including the permutation-based statistical tests for SOMA and the likelihood method would increase their runtime by a factor of greater than 100. The complexity analysis refers to map-to-sequence glocal alignment per map, where *n* is the total length of the *in silico* maps ($\thicksim $500,000 fragments for the human genome), *m*≪*n* is the length of the experimental map in fragments (typically 17 fragments on average), *#seeds*, *c* (default of two) and *δ* are as defined in the “Methods” section and *#it* (number of iterations), *#hashes* (geometric hashes found to match) and |*HashTable*| are as specified in [17, 24]

### Real data analysis and comparison

To assess OPTIMA’s performance on real data we generated, in-house, 309,879 and 296,217 optical maps for two human cell lines, GM12878 and HCT116, respectively, using the Argus system from OpGen [4, 25] (with the *Kpn*I enzyme and ten map cards in total), and glocally aligned them over the human reference genome. Supplementary Note 4 in Additional file 1 provides the sizing error statistics.

Tables 3 and 4 report statistics of the alignments for raw maps filtered under two settings:

(r) relaxed filtering, which filters maps with fewer than ten fragments and smaller than 150 kbp;

**Table 3:** Statistics for glocal alignment of real human optical maps from GM12878 HapMap cell line

Map card	F	Input maps	Details	OPTIMA	Gentig v.2	Increase w.r.t. Gentig v.2	Yield (genome coverage)	Avg. length and size	Avg. digestion rate	Avg. false/extra cut rate	Avg. WHT chi square sizing error
21157LB	(r)	73,365 (7.2X)	Avg. quality 0.50; 295 kbp, 18 f; AFS 16.5 kbp	25 %	9 %	3X	2X	21 f | 324 kbp	66 %	0.74	–0.69
	(s)	38,483 (4.7X)	Avg. quality 0.53; size 368 kbp, 22 f; AFS 17 kbp	36 %	14 %	2.6X	1.7X	23 f | 361 kbp	65 %	0.73	–0.58
21159LB	(r)	75,761 (7.6X)	Avg. quality 0.47; size 300 kbp, 17 f; AFS 17.4 kbp	19 %	5 %	4X	1.6X	19 f | 325 kbp	63 %	0.72	–1.07
	(s)	41,236 (5.1X)	Avg. quality 0.50; size 370 kbp, 21 f; AFS 17.8 kbp	27 %	8 %	3.4X	1.3X	21 f | 359 kbp	62 %	0.72	–0.97
21431LB	(r)	93,896 (8.6X)	Avg. quality 0.52; size 274 kbp, 17 f; AFS 15.8 kbp	20 %	8 %	2.6X	1.9X	21 f | 305 kbp	68 %	0.77	–0.42
	(s)	43,667 (5.1X)	Avg. quality 0.54; size 348 kbp, 21 f; AFS 16.3 kbp	30 %	13 %	2.4X	1.5X	23 f | 343 kbp	67 %	0.77	–0.29
21443LB	(r)	66,857 (6X)	Avg. quality 0.51; size 271 kbp, 17 f; AFS 15.8 kbp	19 %	7 %	2.7X	1.3X	20 f | 299 kbp	67 %	0.77	–0.50
	(s)	29,991 (3.5X)	Avg. quality 0.53; size 346 kbp, 21 f; AFS 16.3 kbp	29 %	12 %	2.5X	1X	23 f | 340 kbp	66 %	0.77	–0.35
TOTAL	(r)	309,879 (29.4X)	Avg. quality 0.50; size 285 kbp, 17 f; AFS 16.4 kbp	21 %	7 %	2.9X	6.8X	21 f | 314 kbp	66 %	0.75	–0.66
	(s)	153,377 (18.3X)	Avg. quality 0.52; size 359 kbp, 21 f; AFS 16.9 kbp	31 %	11 %	2.7X	5.5X	23 f | 352 kbp	65 %	0.75	–0.55

Statistics are reported independently for each map card of GM12878 cell line, using: (r) relaxed filtering: ≥ 10 fragments and 150kbp; and (s) stringent filtering: ≥ 12 fragments and 250kbp (as shown in column F). From left to right are reported: the total number of input maps and their coverage in bases of the human genome; further details such as average map quality (provided by the Argus machine), average map size in bases and length in fragments, and average fragment size (AFS); aligned maps by OPTIMA and Gentig v.2; OPTIMA alignment rate increase with respect to Gentig v.2; other OPTIMA alignment statistics

**Table 4:** Statistics for glocal alignment of real human optical maps from HCT116 colorectal cancer cell line

Map card	F	Input maps	Details	OPTIMA	Gentig v.2	Increase w.r.t. Gentig v.2	Yield (genome coverage)	Avg. length and size	Avg. digestion rate	Avg. false/extra cut rate	Avg. WHT chi square sizing error
17182LA	(r)	10,911 (0.9X)	Avg. quality 0.33; size 257 kbp, 16 f; AFS 15.7 kbp	4 %	0.5 %	8.1X	0.04X	19 f | 245 kbp	66 %	1.29	–1.15
	(s)	3,744 (0.4X)	Avg. quality 0.33; size 351 kbp, 20 f; AFS 17.7 kbp	4 %	0.9 %	4.5X	0.02X	22 f | 326 kbp	63 %	1.23	–0.83
17184LA-2	(r)	55,719 (5.7X)	Avg. quality 0.43; size 305 kbp, 19 f; AFS 16.3 kbp	18 %	9 %	1.9X	1.1X	23 f | 332 kbp	68 %	0.76	–0.65
	(s)	28,658 (3.7X)	Avg. quality 0.45; size 390 kbp, 23 f; AFS 17.2 kbp	25 %	15 %	1.6X	0.9X	25 f | 378 kbp	67 %	0.74	–0.51
17185LA	(r)	56,879 (5.4X)	Avg. quality 0.55; size 285 kbp, 19 f; AFS 14.7 kbp	24 %	18 %	1.4X	1.5X	23 f | 325 kbp	70 %	0.76	–0.17
	(s)	28,003 (3.4X)	Avg. quality 0.59; size 365 kbp, 24 f; AFS 15.1 kbp	35 %	28 %	1.2X	1.2X	26 f | 367 kbp	70 %	0.74	–0.04
17186LA-3	(r)	52,984 (5.8X)	Avg. quality 0.54; size 328 kbp, 20 f; AFS 16.0 kbp	33 %	19 %	1.7X	2X	24 f | 342 kbp	70 %	0.68	–0.35
	(s)	31,588 (4.3X)	Avg. quality 0.56; size 404 kbp, 25 f; AFS 16.4 kbp	42 %	28 %	1.5X	1.7X	26 f | 380 kbp	69 %	0.67	–0.26
17187LA	(r)	88,730 (7.8X)	Avg. quality 0.45; size 264 kbp, 18 f; AFS 14.8 kbp	12 %	7 %	1.7X	1X	21 f | 285 kbp	69 %	0.94	–0.56
	(s)	36,018 (4.2X)	Avg. quality 0.46; size 349 kbp, 22 f; AFS 15.8 kbp	17 %	11 %	1.6X	0.7X	24 f | 338 kbp	68 %	0.92	–0.35
14593LB	(r)	30,994 (2.7X)	Avg. quality 0.39; size 261 kbp, 14 f; AFS 18.9 kbp	6 %	0.6 %	9.9X	0.2X	16 f | 269 kbp	63 %	0.85	–1.23
	(s)	10,944 (1.2X)	Avg. quality 0.39; size 337 kbp, 17 f; AFS 20.2 kbp	9 %	0.7 %	12.3X	0.1X	18 f | 320 kbp	60 %	0.87	–0.97
TOTAL	(r)	296,217 (28.3X)	Avg. quality 0.47; size 287 kbp, 18 f; AFS 15.7 kbp	18 %	11 %	1.7X	5.7X	23 f | 322 kbp	69 %	0.77	–0.44
	(s)	138,955 (17.2X)	Avg. quality 0.50; size 372 kbp, 23 f; AFS 16.5 kbp	27 %	18 %	1.5X	4.6X	25 f | 368 kbp	68 %	0.75	–0.28

Statistics are reported for each map card of HCT116 cell line using the relaxed filtering (r) and the stringent filtering (s), similarly as in Table 3 These results further suggest a mean yield of 1.25 × and 1 × for (r) and (s), respectively, in terms of aligned coverage of the human genome per map card using OPTIMA
(s) stringent filtering (as suggested in [4]), which filters maps with fewer than 12 fragments and smaller than 250 kbp.

The statistics were reported independently for each map card to capture the variability in terms of quality. In total, OPTIMA, with a stringent uniqueness threshold of 30, confidently aligned nearly three times as many maps as Gentig (with default parameters) for GM12878. Similarly, for HCT116, OPTIMA results were 1.7 times better than Gentig results, and corresponding improvements were also obtained using the stringently filtered datasets.

Further analyzing the error rates in the maps that OPTIMA confidently aligned (Tables 3 and 4), we observed that the overall statistics for average digestion rate *d*, false/extra cut rate *f*_100_ and sizing errors were found to be similar to those obtained using scenario (B) (see Supplementary Note 5 in Additional file 1).

### Overlap alignment results

For overlap alignment, we compared OPTIMA-Overlap with an overlap-finding extension of Gentig v.2 (implemented in the commercial software Genome-Builder from OpGen, which contains a module called SCAFFOLDEXTENDER) [17, 24], as well as with Valouev’s likelihood-overlap method [13].

In our first test, we randomly selected 1000 maps for each scenario (A) and (B) from our previously simulated maps for *Drosophila* (BDGP 5) and human (hg19/GRCh37) genomes. In addition, we simulated assembled sequence fragments for these genomes based on empirically derived scaffold size distributions (*Drosophila* assembly N50 of 2.7 Mbp with 239 scaffolds and human assembly N50 of 3.0 Mbp with 98,987 scaffolds); the simulated assemblies were used to generate *in silico* maps (filtered for those with fewer than four non-end fragments, because these cannot be confidently aligned [14, 15]), which were then aligned with the simulated experimental maps.

For our second test, we compared all methods on optical mapping data generated in-house from the K562 human cancer cell line on OpGen’s Argus system, where a random sample of 2000 maps with at least ten fragments was extracted. *In silico* maps were generated from *de novo* assemblies of shotgun Illumina sequencing data (HiSeq) and six mate-pair libraries with insert sizes ranging from 1.1 kbp to 25 kbp [26] (the final assembly had an N50 of 3.1 Mbp and 76,990 scaffolds in total, using SOAPdenovo v.1.05 [27] with a *k*-mer size of 51 for contig assembly and Opera v.1.1 [28] for scaffolding with mate pairs). It is likely that this dataset represents a harder scenario, with assembly gaps/errors and genomic rearrangements confounding the analysis. It also represents a likely use case where mapping data will be critical to detect large structural variations, disambiguate complex rearrangements and, ultimately, assemble cancer genomes *de novo*.

**Fig. 5:**
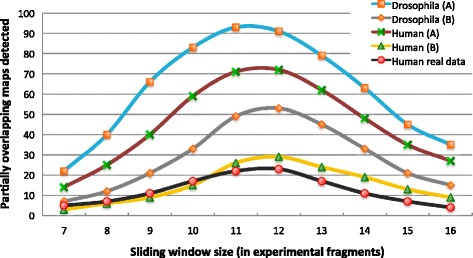
Trade-off for partial overlap detection. Number of (correct) partial overlaps found for each sliding-window size using OPTIMA-Overlap, for both simulated (*Drosophila* and human scenarios (*a*) and (*b*)) and real maps over simulated and real scaffolds (K562 human cancer cell line), respectively

For each test, we evaluated the precision of alignments as well as the number of (correctly) reported alignments that provide an extension to the *in silico* maps through experimentally determined fragments, as this is key for the application of overlap alignments in genome assembly. We begin by noting that there is an important trade-off between sensitivity with a specific window size in OPTIMA-Overlap and the correctness of alignments, as can be seen in Fig. 5. As expected, even though small window sizes (less than ten in Fig. 5) provide more sensitive results, they also make true alignments indistinguishable from noise and reduce the number of correct alignments detected; on the other hand, larger window sizes improve the signal-to-noise ratio but result in a drop in sensitivity. This leads to a sweet spot in the middle (10–13 fragments) where the method is most sensitive across a range of datasets. In particular, real datasets are slightly more challenging than our simulations (see human (B) compared to real data in Fig. 5) and so we have conservatively chosen a window size of 12 as the default for OPTIMA-Overlap. By benchmarking OPTIMA-Overlap with this setting, we observed high precision similar to that observed with OPTIMA for glocal alignment (Table 5). This was seen uniformly across datasets with disparate profiles in terms of genome size and error rates, suggesting that our statistical evaluation is reasonably robust. As before, we also note that Gentig’s approach and the likelihood-based method might not always exhibit high precision. Finally, in terms of sensitivity, OPTIMA-Overlap shows a 30–80 % improvement over competing approaches, and this is also seen in the harder real datasets.

**Table 5:** Comparison of methods for overlap map-to-sequence alignment

Algorithm	*Drosophila* (A)		*Drosophila* (B)		Human (A)		Human (B)		Human real data
	E	P	E	P	E	P	E	P	E
OPTIMA-overlap	**91**	**100**	**53**	**98**	**72**	**99**	**29**	**97**	**23**
Gentig v.2 (d)	69	100	29	93	51	93	19	83	14
Likelihood-overlap (d + a)	59	74	36	52	21	41	9	26	12

The precision of overlap alignments (P, in percentages) and the number of overlap alignments that lead to (correct) extensions (E, absolute values) as a measure of sensitivity (correctness is only known for simulated datasets) are shown. The best values across methods are highlighted in bold

### Utility in real-world applications

Overlap alignments form a critical building block for applications such as OpGen’s Genome-Builder and its use in boosting assembly quality [4]. As OPTIMA-Overlap can work with lower quality data (scenario (B) in our simulations; Genome-Builder would typically filter out such data) and also provide improved sensitivity in detecting overlap alignments, we estimate that its use could reduce the requirement for generating mapping data by half. As the cost of mapping data for the assembly of large eukaryotic genomes can range from USD 20,000 to 100,000, this can lead to significant cost savings.

We similarly compared OPTIMA and Gentig on the two human cell line results, shown in Tables 3 and 4, in order to calculate how much mapping data would be needed for sufficient aligned coverage of the human genome to enable structural variation analysis. By analyzing the alignment rate increase of OPTIMA compared to Gentig, a 1.5 to 2.9 times increase on average, we computed the corresponding cost reduction to be 33–66 %, with an average cost reduction of 54 % for relaxed filtering of data (r) and 49 % for stringent filtering (s). These results suggest that for structural variation analysis on the human genome, particularly for cancer genomes, OPTIMA can significantly reduce project costs (in the tens of thousands of dollars) while enabling faster analyses of the data.

## Conclusion

With the availability of new mapping technologies (for example, Nabsys) and greater use of existing ones to complement high-throughput sequencing, there is a critical need for robust computational tools that can combine genomic mapping and sequence data efficiently. In this work, we introduce two new alignment tools that address this need for a wide range of applications, from genome assembly to structural variation analysis. Our benchmarking results provide evidence that these methods outperform existing approaches in sensitivity and runtime while providing highly precise alignments in a range of experimental settings. Similar results are also seen in real datasets from human cell lines, suggesting that they could help in significantly reducing the cost of optical mapping analysis and thus increase its usage.

In the development of OPTIMA and OPTIMA-Overlap, we establish two key new ideas for map alignment. The first is the introduction of composite seeds, an idea that echoes the idea of spaced seeds in the context of continuous-valued sequence alignment. Composite seeds allow us to develop efficient seed-and-extend aligners for map-to-sequence alignment of erroneous mapping data. We believe that similar ideas can be applied for map-to-map alignment and *de novo* assembly of experimental maps. The second concept is the development of a conservative statistical testing approach that does not require knowledge of the true distribution of errors or an expensive permutation test to evaluate the uniqueness and significance of alignments. This allows us to significantly reduce the runtime cost of this step without sacrificing specificity or the ability to be agnostic with respect to error rates. Although our experiments with real data in this work were limited to data generated on the Argus system from OpGen, similar ideas (with minor variations) should also be applicable to data generated by other technologies such as the Irys platform from BioNano Genomics.

In future work, we plan to implement further runtime and memory optimizations to OPTIMA and OPTIMA-Overlap and to explore their use for super-scaffolding of large genomes as well as for studying genomic rearrangements in cancer.

## Availability and requirements

- **Project name:** OPTIMA: Index-based map-to-sequence alignment in large eukaryotic genomes
- **Project home page:**http://www.davideverzotto.it/research/OPTIMA, https://github.com/verznet/OPTIMA
- **Operating system:** Platform independent
- **Programming language:** Java 7+
- **Other requirements:** Java Development Kit 7+, Apache Commons Math 3.2, CERN Colt 1.2.0
- **License:** LGPL (Lesser General Public License) 2.1, OSI compliant
- **Any restrictions to use by non-academics:** none

## Availability of supporting data

Snapshots of the code and benchmarking and real datasets are available from the *GigaScience* GigaDB database [29, 30].

## Additional file

Additional file 1 **Supplementary material.** Supplementary notes and figures to accompany the main manuscript. (PDF 32358 kb)
