# Supplementary material for: OPTIMA: sensitive and accurate whole-genome alignment of error-prone genomic maps by combinatorial indexing and technology-agnostic statistical analysis
Source: Gigascience. 2016 Jan 19;5:2. doi: 10.1186/s13742-016-0110-0 (PMC4719737; doi:10.1186/s13742-016-0110-0)
Supplement: Supplementary file 1 — Supplementary material. Supplementary notes and figures to accompany the main manuscript. (PDF 32358 kb) [file 13742_2016_110_MOESM1_ESM.pdf]

RESEARCH

Supplementary Material to: OPTIMA: Sensitive and accurate whole-genome alignment of error-prone genomic maps by combinatorial indexing and technology-agnostic statistical analysis

Davide Verzotto[1*], Audrey SM Teo[2], Axel M Hillmer[2] and Niranjan Nagarajan[1*]

*Correspondence: {verzottod,nagarajann}@gis.a-star.edu.sg
[1]Computational and Systems Biology, Genome Institute of Singapore, 60 Biopolis Street, 138672 Singapore, Singapore
Full list of author information is available at the end of the article

Supplementary Note 1 – Banded dynamic programming

When some thresholds on the number of missing and false cuts are known *a priori*, or are provided, we can band the dynamic programming as follows.

Let us suppose that in the worst case the average cut digestion is d, the average false/extra cut rate is f_{100} per 100 kilo base pairs (kbp) and the average fragment size is denoted by AFS; then:

$$\mathrm{P}(> j \text{ missing cuts in a row}) = (1-d)^{j+1}, \tag{1}$$

and

$$\mathrm{P}(> i \text{ false cuts in a row}) \approx (f_{100}/100000 \times \mathrm{AFS})^{i+1}. \tag{2}$$

For example, if $d = 0.61$, $f_{100} = 1.38$ false cuts per 100 kbp and AFS $= 10.8$ kbp (the harder scenario (B) presented in the Results and discussion section of the main manuscript [1]), then:

$$\mathrm{P}(> 8 \text{ missing cuts in a row}) \approx 10^{-4},$$

that is, one case with more than eight missing cuts in a row every 100 Mbp, on average, and:

$$\mathrm{P}(> 5 \text{ false cuts in a row}) \approx 10^{-5},$$

that is, one case with more than five false cuts in a row every 1 Gbp, on average. These parameters – maximum eight missing cuts and maximum five false cuts in a row – can be used to define the boundaries of the dynamic programming and also to limit the backtracking while maintaining good alignment sensitivity in large eukaryotic genomes. Clearly, if more accurate datasets are provided, tighter values based on Equation 1 and Equation 2 can be used to increase the speed of computation. For instance, in scenario (A) of the Results and discussion section it would be sufficient to limit missing and false cuts in a row to six and four, respectively.

Supplementary Note 2 – Details of OPTIMA's scoring function

In this framework, matches comprising the ends of the experimental map or the *in silico* maps count towards the total number of cut errors ($\#cuterrors$) but not towards the total χ^2 computed for the entire glocal alignment. Once a glocal alignment is computed, we remove the penalty for very small fragments (below 800 bp) that are either missing fragments or characterize missing cuts inside feasible matches.

In addition, in cases of short *in silico* maps, we allow the system to learn the null model from a bigger space, by concatenating the *in silico* maps or by giving as input *in silico* maps of similar genomes.

Supplementary Note 3 – Parameter settings for Gentig, SOMA and the likelihood-based method

By default, Gentig discards very small *in silico* fragments below 400 bp and sets $d = 0.85$ and $f_{100} = 0.5$; SOMA discards *in silico* fragments below 800 bp; and the likelihood-based method discards *in silico* fragments below 2 kbp and sets its internal parameters function to $sp(\delta = 5, \lambda = \mathrm{AFS}, \sigma = 0.579, f_1 = 0.005, d = 0.8, \eta = 3, \Delta = 1\,\mathrm{kbp})$ (as defined in [2]). For Valouev's likelihood-based (p), we set the parameters function to $sp(\delta = 7, \lambda = \mathrm{AFS}, \sigma = 0.553, f_1 = f_{100}/100, d, \eta = 2.236, \Delta = 4\,\mathrm{kbp})$.

Supplementary Note 4 – Sizing errors in real data

Real OpGen data were first analyzed to obtain a model for sizing errors in OPTIMA (as well as to obtain parameters for Gentig, using a proprietary script from OpGen). We identified *a posteriori* the following sizing error model (computed from reference fragment sizes, r):

$$\text{mean} = \max \begin{cases} -0.025\,r + 100\,\mathrm{bp} \\ -400\,\mathrm{bp} \end{cases},$$
$$\text{standard deviation} = 0.03\,r + 450\,\mathrm{bp}. \tag{3}$$

By evaluating this model on one-to-one experimental–*in silico* fragment matches, we confirmed our assumption that fragment sizes of experimental data generated by the Argus system approximately follow a normal distribution, which is consistent across different map cards and cell lines (see Q–Q plots of Supplementary Figure 1A and Supplementary Figure 1B). Supplementary Figure 1C also shows that the mean of sizing errors is in general not zero and varies with each sizing class. Finally, experimental optical maps are typically 1.5–2% smaller than their corresponding *in silico* reference regions.

Supplementary Note 5 – Concordance between synthetic and real data

By glocally aligning the synthetic data of scenario (B) on the human reference genome, we obtained the following average values for the relaxed scenario (r): $d = 69\%$, $f_{100} = 0.98$ and $\mathrm{WHT}(\chi^2, \#matches) = -1.52$. These values approximately fit the average values obtained with real data shown in Table 3 and Table 4 of [1].

We can further observe an additional alignment rate reduction of 51% (from 43% to 21%) and 58% (from 43% to 18%) for GM12878 and HCT116 cell lines, respectively, in the relaxed scenario (r).

Author details
[1]Computational and Systems Biology, Genome Institute of Singapore, 60 Biopolis Street, 138672 Singapore, Singapore. [2]Cancer Therapeutics and Stratified Oncology, Genome Institute of Singapore, 60 Biopolis Street, 138672 Singapore, Singapore.

References
1. Verzotto D, Teo ASM, Hillmer AM, Nagarajan N. OPTIMA: Sensitive and accurate whole-genome alignment of error-prone genomic maps by combinatorial indexing and technology-agnostic statistical analysis. GigaScience. 2016;4:65.
2. Valouev A, Li L, Liu YC, Schwartz DC, Yang Y, Waterman MS, et al. Alignment of Optical Maps. Journal of Computational Biology. 2006;13:442–462.

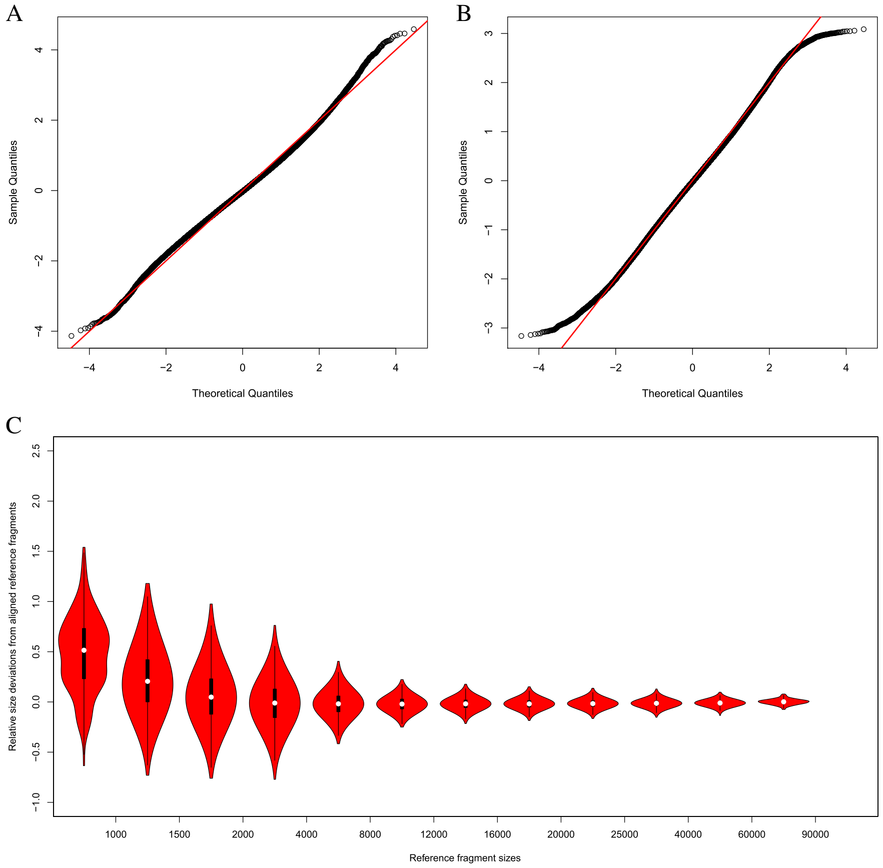

Supplementary Figure 1. Normal Q–Q plots of the preprocessed sizing error model applied to optical mapping data, and corresponding violin plots. A Normal Q–Q plot for sizing errors from optical maps of GM12878 human HapMap cell line. **B** Normal Q–Q plot for sizing errors from optical maps of HCT116 human colorectal cancer cell line. These Q–Q plots, based on an ensemble of multiple map cards and one-to-one experimental–reference fragment matches, indicate the approximate correspondence of normalized sizing errors with the standard normal distribution (the ideal curve is represented in red). **C** Violin plots (for HCT116 17186LA-3 map card) for relative deviations of different classes of fragment size. The figure emphasizes that there is a higher spread for fragments below 4 kbp in real data.
